# Ultrasonic Treatment Enhances the Antioxidant and Immune-Stimulatory Properties of the Polysaccharide from *Sinopodophyllum hexandrum* Fruit

**DOI:** 10.3390/foods12050910

**Published:** 2023-02-21

**Authors:** Ziwei Liu, Hangyu Li, Qianqian Liu, Yangyang Feng, Daiyan Wu, Xinnan Zhang, Linzi Zhang, Sheng Li, Feng Tang, Qun Liu, Xiaonong Yang, Haibo Feng

**Affiliations:** 1 College of Animal Husbandry and Veterinary Medicine, Southwest Minzu University, Chengdu 610041, China; 2 Key Laboratory of Ministry of Education and Sichuan Province for Qinghai-Tibetan Plateau Animal Genetic Resource Reservation and Utilization, Chengdu 610041, China

**Keywords:** ultrasonic, carbohydrate, structural characterization, antioxidant, immunomodulatory effect

## Abstract

We aimed to assess the potential of ultrasonic treatment on the processing of polysaccharides as functional foods or food additives. The polysaccharide from *Sinopodophyllum hexandrum* fruit (SHP, 52.46 kDa, 1.91 nm) was isolated and purified. SHP was treated with various levels of ultrasound (250 W and 500 W), resulting in the formation of two polysaccharides, SHP1 (29.37 kD, 1.40 nm) and SHP2 (36.91 kDa, 0.987 nm). Ultrasonic treatment was found to reduce the surface roughness and molecular weight of the polysaccharides, leading to thinning and fracturing. The effect of ultrasonic treatment on polysaccharide activity was evaluated in vitro and in vivo. In vivo experiments showed that ultrasonic treatment improved the organ index. Simultaneously, it enhanced the activity of superoxide dismutase, total antioxidant capacity, and decreased the content of malondialdehyde in the liver. In vitro experiments demonstrated that ultrasonic treatment also promoted proliferation, nitric oxide secretion, phagocytic efficiency, costimulatory factors (CD80+, CD86+) expression, and cytokine(IL-6, IL-1β) production of RAW264.7 macrophages.

## 1. Introduction

Plant polysaccharides are carbohydrates comprising many different kinds of monosaccharides. They perform biological activities such as immune regulation as well as anti–inflammatory and anti–viral actions. Currently, polysaccharides have become a hot spot in food science because of their immense potential and economic value [1].

*Sinopodophyllum hexandrum* is a plant that is mainly distributed in Yunnan, Sichuan, Tibet and other places in China [2]. Its fruit appears as a red oval when it matures. Local people usually pick and eat this fruit directly. At this time, research in China and abroad focuses on podophyllotoxin extracted from the root of this plant, and there is little research on its fruit [3]. The polysaccharide found in the fruit of SHP is a type of biological macromolecule with antioxidant and immune regulation properties, and it has great potential to become a food additive. Due to the high molecular weight of the extracted polysaccharide, it is difficult for it to exert its biological activity, and some special methods are required to degrade the molecular weight of the polysaccharide [4,5,6,7,8]. At present, there are three known methods of polysaccharide degradation, namely, biological degradation, chemical degradation, and physical degradation. Enzymatic degradation is the most attractive type of biodegradation. Enzymatic degradation of polysaccharides makes use of its specificity and non–specificity and will not damage the effective functional groups of the substrate and the structure of the oligosaccharide itself in the reaction process, so the activity of the product is relatively high. In addition, the molecular weight of the degradation products is easy to control, the reaction conditions are mild, and the pollution is relatively small. However, the cost of enzyme degradation is too high to be widely used in industry. The chemical degradation of polysaccharides mainly uses chemical reagents such as NaNO_2_, acid, and oxidant to degrade polysaccharides. The advantages of this treatment method are low cost and rapid reaction, but the homogeneity of degradation products is relatively poor, the activity of polysaccharides will be broken, and the residues of these reagents will also lead to environmental pollution [9]. Physical degradation is a green and efficient degradation method, including microwave, ion radiation and high–pressure micro jet. These methods are difficult to operate and difficult to promote in the food industry. Therefore, finding a new and harmless way to degrade the molecular weight of polysaccharides is of great significance for polymers such as polysaccharides to become functional foods or additives in the food industry. In the past 10 years, the ultrasonic–assisted method has been widely used in the extraction of polysaccharides. Some studies have shown that ultrasonic improves the extraction rate of polysaccharides. The ultrasonic–assisted method uses the cavitation, mechanical, and thermal effects of ultrasonic to extract the effective components from raw materials [10,11,12]. Ultrasonic treatment also significantly enhanced the efficiency of polysaccharide extraction compared with the use of reagents only. Studies have shown that ultrasound can also be used for polysaccharide degradation. Both the process and extent of ultrasonic degradation can be easily controlled, resulting in the production of structurally intact low molecular-weight polysaccharides without the necessity of introducing new substances. Selection of the specific ultrasonic treatment leads to effective cleavage of the macromolecular chain and polysaccharide degradation [9].

The activity of polysaccharides is determined by the active centres of several oligosaccharide fragments in the polysaccharide molecule. Through ultrasonic degradation, the polysaccharide molecule begins to decompose from the centre. In this process, the physical and chemical properties of polysaccharides change slowly, and their biological activities, such as antioxidant activity and immune activity, also change [13].

Several studies have shown that polysaccharides can enhance the activity of antioxidant enzymes and reduce the levels of oxidative stress. For example, polysaccharides from *Taraxacum mongolicum* and *Cissus pteroclada Hayata* can increase the activity of superoxide dismutase and reduce the malondialdehyde content in the body. In addition, macrophages play a major role in innate immunity. Polysaccharides, such as apple, can enhance the immune function of macrophages [14,15,16]. In order to investigate the effect of ultrasonic treatment on polysaccharide functions, we evaluated the effects of ultrasonic irradiation on the structure and antioxidant activity of *Sinopodophyllum hexandrum* polysaccharide in vivo. Additionally, we conducted in vitro experiments to initially assess the potential toxicity and immunological effects of the ultrasonically treated polysaccharide on cells. We believe that our study results will provide a theoretical basis for the application of ultrasonic in the processing of polysaccharides as functional foods and additives.

## 2. Materials and Methods

### 2.1. Materials and Chemical Reagents

Neutral red was obtained from Leagene Biotechnology Co., Ltd. (Beijing, China). SHP fruit was obtained from Cangxitang Biotechnology Co., Ltd. (Chengdu, China). HCSS was obtained from National Institutes for Food and Drug Control (Beijing, China). LPS was obtained from Solarbio Technology Co., Ltd. (Beijing, China). Fluorescein thiocyanate (FITC) and DAPI (4′,6–diamidino–2–phenylindole) were obtained from Yuanye Biotechnology Co., Ltd. (Shanghai, China); DID (DiIC18(5) (1,1′–dioctadecyl–3,3,3′,3′– tetramethylindodicarbocyanine, 4-chlorobenzenesulfonate salt) was obtained from C–reagent Biotechnology Co., Ltd. (Shanghai, China); and sodium sulphate and sodium azide were purchased from Aladdin Biochemical Technology Co., Ltd. (Shanghai, China). The Nitric Oxide Assay kit was purchased from Yuhengsheng Material Technology Co., Ltd. (Suzhou, China). Superoxide dismutase (SOD), total antioxidant capacity and malondialdehyde (MDA) were obtained from the Nanjing Jiancheng Bioengineering Research Institute (Nanjing, China). The Cell Counting Kit–8, Dulbecco’s Modified Eagle Medium (DMEM), and sterile phosphate-buffered saline (PBS) were provided by Boster Biological Technology Co., Ltd. (Wuhan, China).

### 2.2. Ultrasonic Treatment

Response surface methodology was used to optimize the extraction process of polysaccharides, and this part is presented as Supplementary Material. According to the method described in the literature, polysaccharides were decolorised using macroporous resin and purified using cellulose column, freeze-dried, and named SHP [17]. The polysaccharide solution was treated with ultrasonic at two different power levels (250 W and 500 W) in a specific pulse mode (1 s on and 1 s off) with an ultrasonic cell breaker (IID, Scientz, Ningbo, China) for 1 h. The treated solution was lyophilised to obtain two degraded polysaccharides named SHP1 and SHP2.

### 2.3. Structural Characterization

#### 2.3.1. Determination of Molecular Weight of the Polysaccharide

The molecular weight of polysaccharides was determined by the gel–permeation chromatography (Eleos system, Wyatt, MO, USA) method. We set the temperature of the chromatographic column to 40 °C and injected 500 μL of the sample at a flow rate of 1 mL/min, and the mobile phase was 0.05 mol/L Na_2_SO_4_ and 0.02% NaN_3_.

#### 2.3.2. Nuclear Magnetic Resonance (NMR)

We accurately weighed the 40 mg sample and dissolved it in D_2_O, followed by ^1^H spectra analysis on an AVII–600 MHz nuclear magnetic resonance spectrometer (Bruker, Aarau, Switzerland).

#### 2.3.3. Fourier Transform Infrared Spectroscopy (FT–IR)

We mixed and ground the polysaccharide with potassium bromide, and pressed the tablet and scanned it with a Fourier Transform Infrared Spectrometer (Cary660, Agilent Technologies, Inc., Beijing, China) in the range of 400–4000 cm.

#### 2.3.4. Circular dichroism (CD) Spectra

We used the CD spectrum (J-815, Jasco, Tokyo, Japan) to detect the configuration of polysaccharides in water. After the polysaccharide was fully dissolved in distilled water, it was scanned at 190–400 nm on the machine.

#### 2.3.5. Atomic Force Microscopy (AFM) and Scanning Electron Microscopy (SEM) Tests

AFM was used to study the morphology of polysaccharides. Three kinds of polysaccharides were dripped on the surface of fresh mica, respectively. After they were completely dried, they were placed on AFM (Dimension ICON, Bruker, Aarau, Switzerland) for observation [18]. Finally, the morphological characteristics of the three samples were analysed by the SEM (SU8220, Hitachi, Tokyo, Japan).

### 2.4. In Vivo Antioxidant Test

Forty–eight–week–old female mice (29 ± 3 g) supplied by Dashuo Laboratory Animal Center (Chengdu, China) were housed at 26 ± 2 °C on a regular (12 h/12 h) light/dark cycle and were given free diets for 7 days. After that, the mice were randomly divided into four groups (10 mice in each group). One group was given normal saline by gavage, and the other three groups were given various polysaccharides (300 mg/kg, 0.5 mL/day) by gavage every other day for 1 week. On the 8th day, the thymus, spleen and liver were taken to calculate the organ index. Finally, the liver was ground into tissue homogenate, and the SOD activity, total antioxidant capacity, and MDA content in the tissue homogenate were measured using a kit. In this study, the procedures related to animal care were performed in accordance with the internationally accepted principles as listed in the Guidelines for Keeping Experimental Animals issued by the government of China.

The organ index (‰) was calculated as follows:

organ index (‰) = organ mass (g)/mouse mass (g) × 1000 (1)

### 2.5. Cell Test

#### 2.5.1. Viability Test

The polysaccharide was diluted into five gradients (1000 μg/mL, 500 μg/mL, 250 μg/mL, 125 μg/mL and 62.5 μg/mL) with DMEM and incubated with mouse macrophages (1 × 10^5^/mL) in 96 well plates for 24 h. We added the reagent according to the instructions of CCK-8 kit, and half an hour later, we measured the absorbance at 450 nm with a microplate reader (iMark, BIO-RAD, Hercules, CA, USA). The cell viability was calculated as follows:

Cell viability (%) = (W_1_ − W_0_)/(W_2_ − W_0_) × 100% (2)

where W_1_ is the absorbance of the experimental group, W_2_ is the absorbance of the control group, and W_0_ is the absorbance of the blank group [19].

#### 2.5.2. Nitric Oxide (NO) Secretion Test

We first prepared a density of 1 × 10^5^ macrophage cell suspension. Then, according to the control group (DMEM + macrophages), the positive control group [lipopolysaccharide (LPS) + macrophages], the negative control group [hydrocortisone sodium succinate (HCSS) + macrophages], and the polysaccharide group (macrophages + polysaccharide solutions of different treatments (250 μg/mL).) were added to 24–well plates, 1 mL for each well, with 3 repetitions in each group. After the cells adhered to the wall, the polysaccharide was added for 24 h, the supernatant was collected, and the NO content was obtained according to the method on the NO determination kit.

#### 2.5.3. Neutral Red Phagocytosis Test

Briefly, after the cells adhered to the wall in the 96–well plates, we discarded the medium and added polysaccharides to continue to culture for 24 h, and then removed the medium and added 0.1% (mg/mL) neutral red solution to each well and continued to culture for 30 min. After 10 μL of cell lysate were added to each well, the absorbance was detected at 450 nm with a microplate reader, after 1 h. The phagocytosis rate of cells was calculated as follows:

Phagocytosis rate of cells(%) = W_3_/W_4_ × 100% (3)

where W_3_ is the absorbance of the experimental group, and W_4_ is the absorbance of the control group.

#### 2.5.4. RT–qPCR Test

RT–qPCR was used to detect IL-1β and IL-6. After total RNA was extracted, cDNA was synthesized with a reverse transcription kit and was finally detected by RT–qPCR. The reaction system volume was 25 μL, including 2 μL cDNA, 12.5 μL TB Green^®^ Premix Ex Taq™ II (Tli RNaseH Plus), 0.5 μL ROX Reference Dye, 8 μL sterile water, and 2 μL upstream and downstream primers, as shown in Table 1.

#### 2.5.5. Flow Cytometry Test

Flow cytometry was used to determine macrophage surface costimulators (CD80+, CD86+). In short, three different polysaccharide samples (250 μg/mL) were co–cultured with macrophages for 12 h, cells were collected and stained with anti mouse CD80+ and CD86+ antibodies, and finally analyzed by flow cytometry (CyFlow Cube8, Sysmex Co., Ltd., Berlin, Germany).

#### 2.5.6. Antigen Uptake Capacity Test

At 4 °C, FITC, polysaccharides with different treatments (SHP, SHP1 and SHP2) and ovalbumin (OVA) were dissolved in dimethyl sulfoxide for 13 h, dialyzed with PBS for 3 days, and four different samples (FITC–OVA, FITC–OVA–SHP, FITC–OVA–SHP1 and FITC–OVA–SHP2) were obtained after freeze-drying [20]. Mouse macrophages with a density of 1 × 10^5^ were added to 24–well plates containing round coverslips until the cells were full on the round coverslips. Then, we added different dissolved samples to continue to culture for 12 h, took out the culture medium and fixed the cells with paraformaldehyde (4%); the content of OVA and FITC in each sample was 250 μg/mL. After washing twice with PBS, we performed DAPI staining, washed again with PBS after 10 min, and repeated the above steps for DID staining. Finally, the stained cells were observed using confocal laser scanning microscopy (TSC SP8, ICA, Weztlar, Germany).

## 3. Results and Discussion

### 3.1. Ultrasonic Treatment

**Figure 1:**
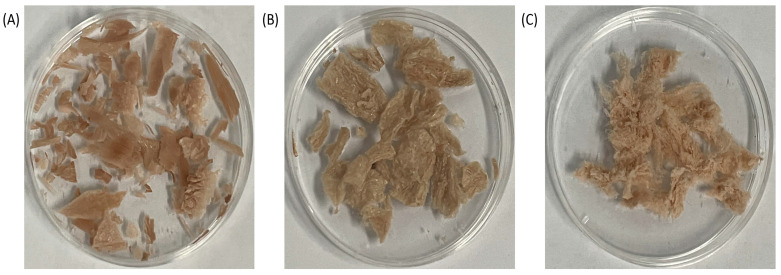
Morphologies of the polysaccharide from *Sinopodophyllum hexandrum* fruit (SHP) and ultrasonic–treated (250 W and 500 W) polysaccharides (SHP1 and SHP2). (**A**) Morphology of SHP. (**B**) Morphology of SHP1. (**C**) Morphology of SHP2.

Analysis using the phenol–sulfuric acid method showed a 7.215% polysaccharide extraction rate after response surface optimization (shown in the Supplementary Material), while the sugar content in SHP could reach 82.1%. The comparative morphology of SHP and the ultrasonic–treated polysaccharides is shown in Figure 1A–C. Compared with SHP, SHP1 appeared fluffier with an absence of lamellar structure. As the ultrasonic frequency increased, SHP2 became gradually fibrotic while retaining a fluffy texture.

### 3.2. Structural Characterization Test

#### 3.2.1. Nuclear Magnetic Resonance (NMR)

**Figure 2:**
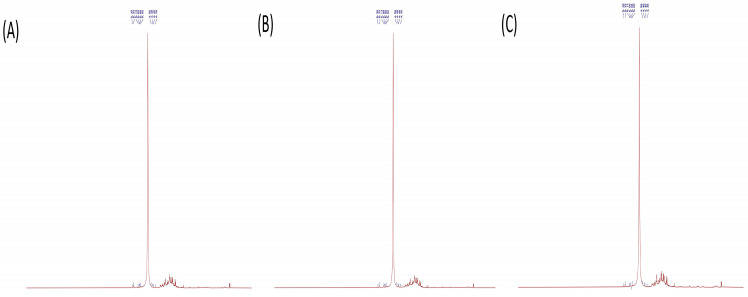
NMR spectra of the polysaccharide from *Sinopodophyllum hexandrum* fruit (SHP) and ultrasonic–treated (250 W and 500 W) polysaccharides (SHP1 and SHP2). (**A**) ^1^H NMR spectra of SHP. (**B**) ^1^H NMR spectra of SHP1. (**C**) ^1^H NMR spectra of SHP2.

In the ^1^H NMR spectrum, δ Values above 5.00 indicate α monosaccharide of configuration, but below 5.00 indicates the presence of β monosaccharides of configuration. As shown in Figure 2A–C, In the ^1^H-NMR spectrum, SHP had significant peaks at 4.00–6.00, which proved that SHP exists at the same time—six α and four β monosaccharides of configuration. After ultrasonic treatment with different powers, we found that each peak value had almost no change, which also means that the corresponding functional groups did not change. Therefore, ultrasonic treatment has no effect on the structure of polysaccharides [21].

#### 3.2.2. Molecular Weight Analysis of the Polysaccharide

**Figure 3:**
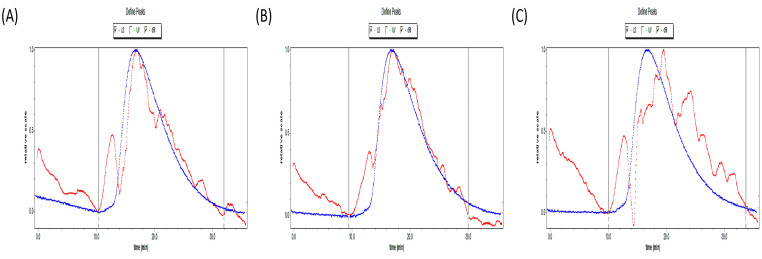
Molecular weight analysis diagram of the polysaccharide from *Sinopodophyllum hexandrum* fruit (SHP) and ultrasonic–treated (250 W and 500 W) polysaccharides (SHP1 and SHP2). (**A**) Molecular weight distribution of SHP. (**B**) Molecular weight distribution of SHP1. (**C**) Molecular weight distribution of SHP2.

As illustrated in Table 2 and Figure 3A–C, the molecular weight of polysaccharides began to decrease after ultrasonic treatment, which confirmed that ultrasonic had the effect of reducing molecular weight. However, in this experiment, the molecular weight of SHP2 was higher than that of SHP1, which might be because there was still a small amount of protein in the purified polysaccharide, the interval of ultrasonic treatment was too short, the metal probe produced an elevated temperature, and the polysaccharide underwent carbonation reaction in aqueous solution. The specific reasons still need to be further studied.

#### 3.2.3. Fourier Transform Infrared Spectroscopy Analysis (FT–IR)

**Figure 4:**
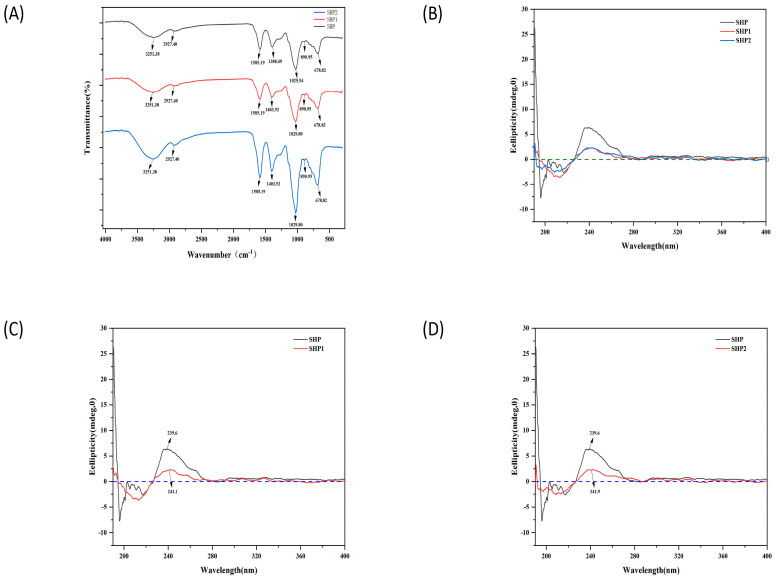
The chemical property of the polysaccharide from *Sinopodophyllum hexandrum* fruit (SHP) and ultrasonic–treated (250 W and 500 W) polysaccharides (SHP1 and SHP2). (**A**) Fourier transform infrared spectroscopy of SHP and ultrasonic–treated polysaccharides. (**B**) Circular dichroism (CD) spectra of SHP and ultrasonic–treated polysaccharides in the wavelength region from 190 to 400 nm. (**C**) CD spectra of SHP and SHP1. (**D**) CD spectra of SHP and SHP2.

The FT–IR spectroscopy of polysaccharides before and after ultrasonic treatment are exhibited in Figure 4A. The absorption peaks of O–H and C–H vibrations were at 3251.38 and 2927.40 cm^−1^, respectively [22]; 1388.49 cm^−1^ (1403.92 cm^−1^) represented the absorption peaks generated by the C–H bending vibration. In addition, the absorption peak at 1025.94 cm^−1^ was attributed to the presence of pyranoside [23]. The absorption peak at 890.95 cm^−1^ indicated the presence of an β–glycoside bond. After ultrasonic treatment, only a small number of values moved slightly to the left after 1500 cm^−1^, and no change was found in each optical energy group. The present results indicated that ultrasonic cannot change the structure of polysaccharides [24].

#### 3.2.4. CD Spectra Analysis

When polysaccharides are dissolved in water, the molecules present irregular forms such as folding and winding, due to the interaction between polysaccharides molecules, resulting in asymmetry and leading to the cotton effect. The CD spectra of ultrasonic-treated and untreated polysaccharides showed a typical cotton effect. From this phenomenon [25], it can be inferred that they all have stable helical tertiary structures. However, the ovality of the CD spectrum and the position of the highest absorption peak of polysaccharide SHP1 and SHP2 changed after ultrasonic treatment (Figure 4B–D). The ellipticity of SHP1 and SHP2 obviously decreased, and the highest absorption peak changed from 193.6 to 241.1 and 241.9, respectively. This may be attributed to the n → π* transition of the carboxyl group [26], and the optical activity of the carboxyl chromophore could be affected by intra- and inter–molecular interactions. After ultrasonic treatment, polysaccharides are degraded to expose more carboxyl groups, resulting in changes in the surrounding environment [27].

#### 3.2.5. AFM and SEM analysis

**Figure 5:**
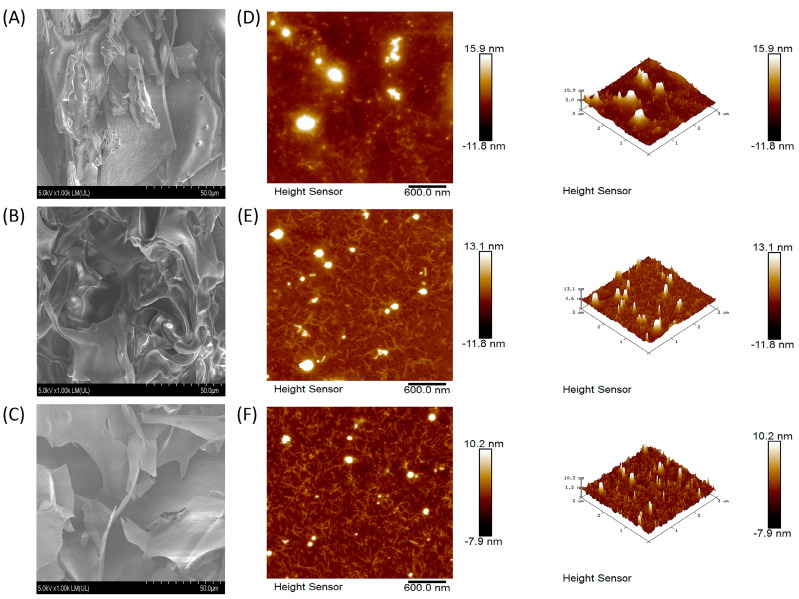
Scanning electron micrographs and atomic force micrographs of the polysaccharide from *Sinopodophyllum hexandrum* fruit (SHP) and ultrasonic–treated (250 W and 500 W) polysaccharides (SHP1 and SHP2). (**A**–**C**) Scanning electron micrographs of SHP, SHP1 and SHP2, (**D**–**F**) atomic force micrographs of SHP, SHP1 and SHP2.

As illustrated in Figure 5A–C, under the scanning electron microscope, the SHP without ultrasonic showed irregular sheet structure, some of which were slightly thicker, and different size pores were observed on the surface and cross–section. Under the ultrasonic treatment of 250 W frequency, the polysaccharide (SHP1) began to thin and gradually became curly like silk. When the ultrasonic frequency reached 500 W, the sheet structure of SHP2 became clearer and thin and gradually began to fracture. The three–dimensional and planar AFM images of SHP are shown in Figure 5D–F. They appeared to consist of an irregular network of fibres arranged in random linear chains, with occasional spherical and non–spherical structures. However, ultrasonic treatment can reduce the surface roughness of polysaccharides. The average roughness of the untreated polysaccharide was 1.91 nm. After 250 W and 500 W ultrasonic treatment, the average roughness decreased to 1.40 nm (SHP1) and 0.987 nm (SHP2), respectively.

### 3.3. In Vivo Antioxidant Test

#### 3.3.1. Organ Index

**Figure 6:**
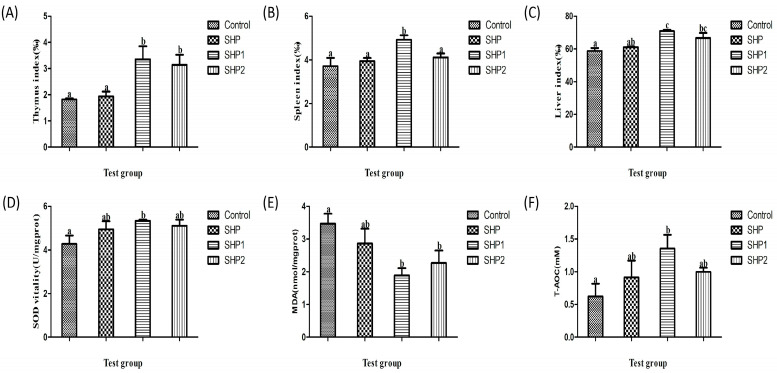
In vivo antioxidant test results. (**A**) Thymus index of mice. (**B**) Spleen index of mice. (**C**) Liver index of mice. (**D**) Superoxide dismutase activity, (**E**) Malondialdehyde content and (**F**) Total antioxidant capacity in mouse liver homogenate. The data are displayed as the mean ± SD (n = 3). The different letters (a–c) designated as statistically significant differences (*p* < 0.05).

The thymus and spleen are the two most important immune organs of the body and play significant roles in the functioning of the immune system. The liver is the principal metabolic organ in the body. It plays an important role in anti–oxidation, detoxification, protein synthesis, and other nutrient functions, and also plays a role in pathogens prevention. As depicted in Figure 6A–C, compared with the control group, the ultrasonic–treated polysaccharides significantly improved the organ indices of the thymus, spleen, and liver in mice (*p* < 0.05). In comparison, SHP1 also led to a marked enhancement in the indices, especially that of the spleen, differing significantly from the other treatment groups (*p* < 0.05). These results indicate the production of lower molecular weight polysaccharides after ultrasonic degradation, which can more easily promote cell proliferation, stimulate the growth of immune organs, and thus improve the immune capacity of the body [28].

#### 3.3.2. Antioxidant Test Analysis

As a common antioxidant enzyme, SOD has a good scavenging effect on reactive oxygen species. MDA is also an oxidation product, which accumulates too much in the body and causes body damage, which is of great significance in the detection of antioxidants [29]. In Figure 6D,E, SHP1 can significantly enhance the SOD activity. There was a significant difference between the SHP1 group and other groups (*p* < 0.05), whereas there was no significant difference between other groups (*p* > 0.05). After ultrasonic treatment, the two polysaccharides significantly reduced the content of MDA in the body, and the MDA content in the SHP1 treatment group was the lowest (*p* < 0.05). In addition, we also tested the total antioxidant capacity. As illustrated in Figure 6F, after intra-gastric administration of SHP1, the total antioxidant capacity of the liver was significantly improved, with an important difference compared with other groups (*p* < 0.05). These results demonstrated that ultrasonic treatment can promote the absorption of polysaccharides, thus enhancing the antioxidant capacity in vivo and improving the immune capacity of the body to a certain extent [30,31].

### 3.4. Cell Test

#### 3.4.1. Analysis of Viability Test

**Figure 7:**
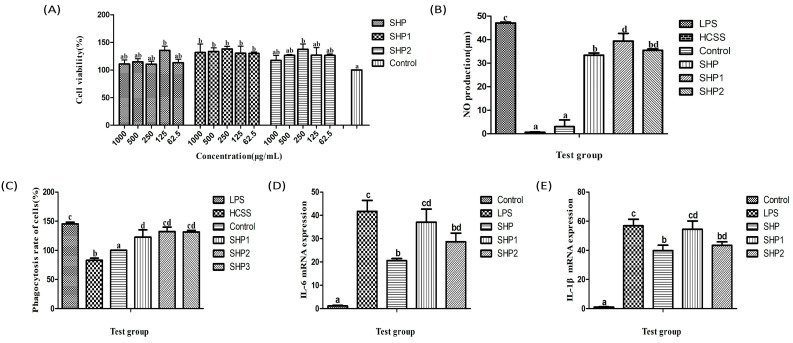
Effect of the polysaccharide from *Sinopodophyllum hexandrum* fruit (SHP) and ultrasonic–treated (250 W and 500 W) polysaccharides (SHP1 and SHP2) on Cell viability, NO secretion and phagocytosis rate of mouse macrophages. (**A**) Proliferation rate, (**B**) NO production, (**C**) Phagocytosis rate, (**D**) IL-6 mRNA expression, (**E**) IL-1βmRNA expression. The data are displayed as the mean ± SD (n = 3). The different letters (a–d) designated as statistically significant differences (*p* < 0.05).

In the immune system, macrophages are the first line of defence, and they participate in almost all immune responses [32]. As shown in Figure 7A, ultrasonic–treated and untreated polysaccharides at concentrations below 1 mg/mL have no toxic effect on mouse macrophages, and both can promote the proliferation of mouse macrophages (*p* < 0.05). There was no significant difference between the concentrations of each polysaccharide group on the effect of cells (*p* > 0.05); thus, the intermediate concentration (250 μg/mL) was selected for the following test.

#### 3.4.2. Analysis of NO Secretion Test

Activated macrophages can inhibit the pathogens by releasing NO, which has a certain bactericidal effect. The results in Figure 7B show that after the addition of polysaccharides, the secretion of NO by mouse macrophages significantly increased, among which the secretion of SHP1 was the highest, with a significant difference compared with SHP (*p* < 0.05) and no significant difference compared with SHP2 (*p* > 0.05).

#### 3.4.3. Analysis of Phagocytosis Test

Neutral red is a fluorescent reagent with a large molecular weight. It can only be ingested into macrophages through endocytosis. After destroying the cells with cell lysate, neutral red inside cells is released, which can be used to analyse the phagocytosis of macrophages. As depicted in Figure 7C, after the addition of polysaccharides, the uptake of neutral red by mouse macrophages increased significantly, with a meaningful difference compared with the control group. The highest phagocytosis rate appeared in the SHP1 group (*p* < 0.05); however, there was no significant difference between the polysaccharide groups (*p* > 0.05).

#### 3.4.4. IL-6 and IL-1β mRNA Expression

IL-6 and IL-1β are important members of the cytokine network and play an important role in immune regulation. As illustrated in Figure 7D,E, SHP can enhance the mRNA expression of IL-6 and IL-1β in macrophages. After ultrasonic treatment, their expression level was further increased (*p* < 0.05), and the expression level of SHP1, which had the smallest molecular weight, was significantly higher than that of other groups (*p* < 0.05). It may be that after the molecular weight of SHP is reduced, it is easy to be used by macrophages.

#### 3.4.5. Expression of Surface Costimulatory Molecules (CD80+, CD86+)

**Figure 8:**
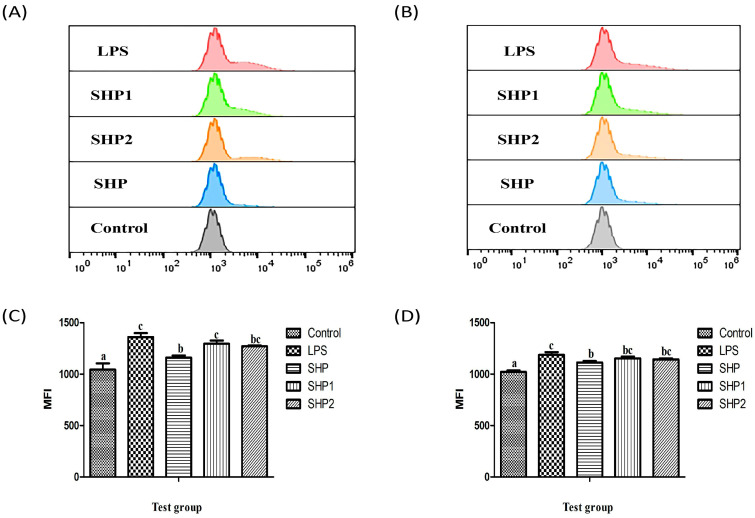
Expression of CD80+ and CD86+ on cell surface of RAW264.7. (**A**) Expression of CD80+. (**B**) Expression of CD86+. The flow cytometry plots of the expression frequencies of CD80+ (**C**) and CD86+ (**D**) in macrophages. The data are displayed as the mean ± SD (n = 3). The different letters (a–c) designated as statistically significant differences (*p* < 0.05).

CD80+ and CD86+ are costimulators when activating T lymphocytes and play a significant part in humoral immune response, transplantation response, and autoimmune monitoring [33]. In addition, they are also typical markers of M1 polarization of macrophages and play an immunomodulatory role by promoting the inflammatory response [34,35]. After ultrasonic treatment, compared with SHP treated macrophages, the expression of CD80+ and CD86+ significantly increased in the macrophage treated by SHP1 and SHP2 (Figure 8A,B, *p* < 0.05). It may be that after the molecular weight of polysaccharide is reduced, it is more easily used by macrophages, more capable of activating cells and increasing the expression of surface costimulatory molecules. The present results indicated that ultrasonic treatment could promote the activity of polysaccharides in macrophage M1 polarization.

#### 3.4.6. Analysis of Antigen Uptake Capacity Test

**Figure 9:**
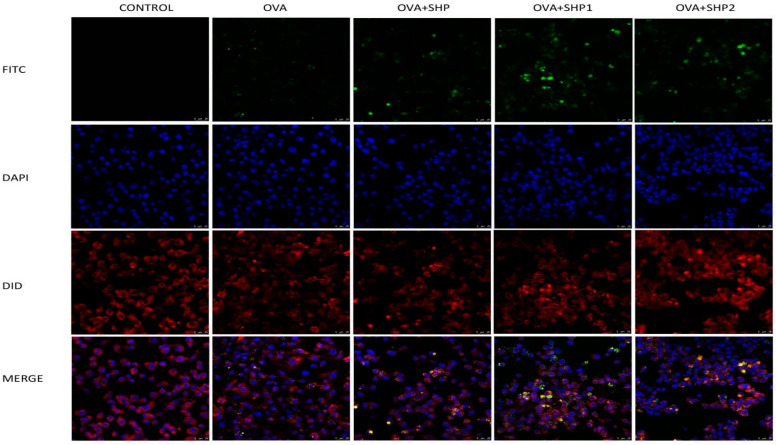
Confocal laser scanning microscopy images about the effect of the polysaccharide from *Sinopodophyllum hexandrum* fruit (SHP) and ultrasonic–treated (250 W and 500 W) polysaccharides (SHP1 and SHP2) on ovalbumin (OVA) phagocytosis of macrophages. The green fluorescence represents FITC labeled OVA, the blue fluorescence represents DAPI stained nuclei, and the red fluorescence indicates DID labeled cell membranes. When the three kinds of fluorescence overlap, they will appear orange.

CLSM was used to observe the uptake of OVA by mouse macrophages. As shown in Figure 9, the green fluorescence intensity was greatly increased after the addition of the polysaccharide and the green fluorescence was strongest after the addition of SHP1. Similarly, after the three fluorescence overlaps, we could see that the number of orange light spots in the polysaccharide groups was increased, which was significantly stronger than was t in the FITC–OVA group, indicating that the uptake of OVA by mouse macrophages was notably increased, and the internalised OVA was mainly distributed in the nucleus. These data demonstrated that both ultrasonic–treated and untreated polysaccharides can enhance the ability of macrophages to phagocytise OVA. Surprisingly, after ultrasonic treatment, the effect of the polysaccharide in promoting cell phagocytosis was significantly enhanced, particularly SHP1, which may be because its molecular weight was the smallest of the three groups of polysaccharides and was easier to be absorbed and utilised. After ultrasonic treatment of polysaccharides, the molecular weight is lower and can be fully utilized by macrophages. After the activation of polysaccharides, macrophages demonstrate stronger antigen phagocytosis ability. It was also confirmed that ultrasonic degradation of polysaccharide molecular weight enhances the biological activity of polysaccharides.

## 4. Conclusions

This study showed that, compared with the naturally extracted polysaccharide, there was no significant change in the main structure of the polysaccharide after ultrasonic treatment. However, ultrasonic treatment led to the production of polysaccharides of lower molecular weight with improved antioxidant and immune–stimulatory capacities. It may be that ultrasonic treatment reduces the thickness of the polysaccharide, together with reduced surface roughness and lower molecular weight with a more extensive contact surface after dissolution, allowing more effective utilization of the polysaccharide. This is the first report of the extraction of this polysaccharide, as well as the first description of the effects of ultrasonic treatment on its activity, showing that ultrasonic treatment improved both immune function in vitro and antioxidant activity in vivo. In summary, ultrasonic treatment is an efficient and non–toxic method for the processing of functional foods or food additives. Nevertheless, more in–depth research is required to explore the mechanism responsible for the degradation of polysaccharides by ultrasonic treatment and whether it produces additional effects on polysaccharides.

## Figures and Tables

**Table 1:** Mouse primer sequences used for qPCR.

Gene Name	Forward Primer (5′→3′)	Reverse Primer (5′→3′)
IL1β	GTGTCTTTCCCGTGGACCTTC	TCATCTCGGAGCCTGTAGTGC
IL6	CTTGGGACTGATGCTGGTGAC	TCTCATTTCCACGATTTCCCAG
GAPDH	GGGTCCCAGCTTAGGTTCATC	TACGGCCAAATCCGTTCACA

**Table 2:** Molecular weight distribution.

Analysis Item	Index	SHP	SHP1	SHP2
Molecular weight	Mp (kDa)	15.74	8.344	12.50
	Mw (kDa)	52.46	29.37	36.91

## Data Availability

The data presented in this study are available on request from the corresponding author.

## Supplementary Materials

The following supporting information can be downloaded at: https://www.mdpi.com/article/10.3390/foods12050910/s1. References [36,37,38,39,40,41] are cited in the supplementary materials.
