# Supplementary material for: Ultrasonic Treatment Enhances the Antioxidant and Immune-Stimulatory Properties of the Polysaccharide from Sinopodophyllum hexandrum Fruit
Source: Foods. 2023 Feb 21;12(5):910. doi: 10.3390/foods12050910 (PMC10001073; doi:10.3390/foods12050910)
Supplement: Supplementary file 1 [file foods-12-00910-s001.zip › foods-2141291-supplementary.pdf]

Supplementary material

Journal name: Foods

Ultrasonic Treatment Enhances the Antioxidant and Immune-Stimulatory Properties of the Polysaccharide from Sinopodophyllum hexandrum Fruit

Ziwei Liu 1,2, Hangyu Li 1,2, Qianqian Liu 1,2, Yangyang Feng 1,2, Daiyan Wu 1,2, Xinnan Zhang 1,2, Linzi Zhang 1,2, Sheng Li 1,2, Feng Tang 1,2, Qun Liu 1,2, Xiaonong Yang 1,2 and Haibo Feng 1,2,*

1 College of Animal Husbandry and Veterinary Medicine, Southwest Minzu University, Chengdu 610041, China

2 Key Laboratory of Ministry of Education and Sichuan Province for Qinghai-Tibetan Plateau Animal Genetic Resource Reservation and Utilization, Chengdu 610041, China

* Correspondence: fenghaiborc@126.com; Tel./Fax: +86-28-85522310

Optimization of the extraction process

We used the method of water extraction and alcohol precipitation to extract the polysaccharides [1]. We ground the dried fruit into a powder, sieved it, accurately weighed 10 g in a round bottom flask, added 95% ethanol (1:20), and removed the fat using a rotary evaporator (RE-2000B, Yarong Biochemical Instrument Co., Ltd., Shanghai, China). We used distilled water as an extraction solvent and the extraction was performed according to a certain liquid solid ratio, extraction time, and extraction temperature. After filtering out the residue, we concentrated the liquid to 50 mL with a rotary evaporator, added thrice the volume of ethanol (95%), and stayed at 4℃ overnight. The precipitate was placed in a centrifuge tube, which was centrifuged to remove the supernatant. Finally, the alcohol sediment was freeze-dried to obtain crude polysaccharides. Glucose standard curves were prepared according to the methods described in the literature, and the extraction rate of polysaccharides was calculated [2].The regression equation thus obtained was as follows: $Y = 0.0112 x + 0.0751$, $R^2 = 0.9993$. The extraction rate of polysaccharides was as follows: $W = C \times V \times N / m$, where W is the polysaccharide, C is the polysaccharide concentration of

the extract (mg/mL), V is the extraction volume (mL), N is diluted multiples and m is the quality of dry powder (g) in a single-factor experiment.we set the single factor test factors: extraction time, extraction temperature, and liquid material ratio. The influence of the single factor on the extraction rate of polysaccharides was investigated under the condition that the two factors remained unchanged. The single factor test design is shown in Table S1. According to the single factor experimental results, the extraction time, liquid material ratio, and extraction temperature were designed as independent variables, whereas the polysaccharide extraction rate was the response value. The response surface factor levels are shown in Table S2.

Table S1 Single factor test design

Extraction time(h)	Extraction temperature(℃)	Liquid material ratio(mL/g)
1	60	10:1
2	70	20:1
3	80	30:1
4	90	40:1
5	100	50:1

Table S2 Response surface test factor levels

Levels	Factors		
	Extraction Time(h)	Extraction temperature(℃)	Liquid material ratio(mL/g)
-1	3	80	10:1
0	4	90	20:1
1	5	100	30:1

Results and discussion

Process optimization test analysis

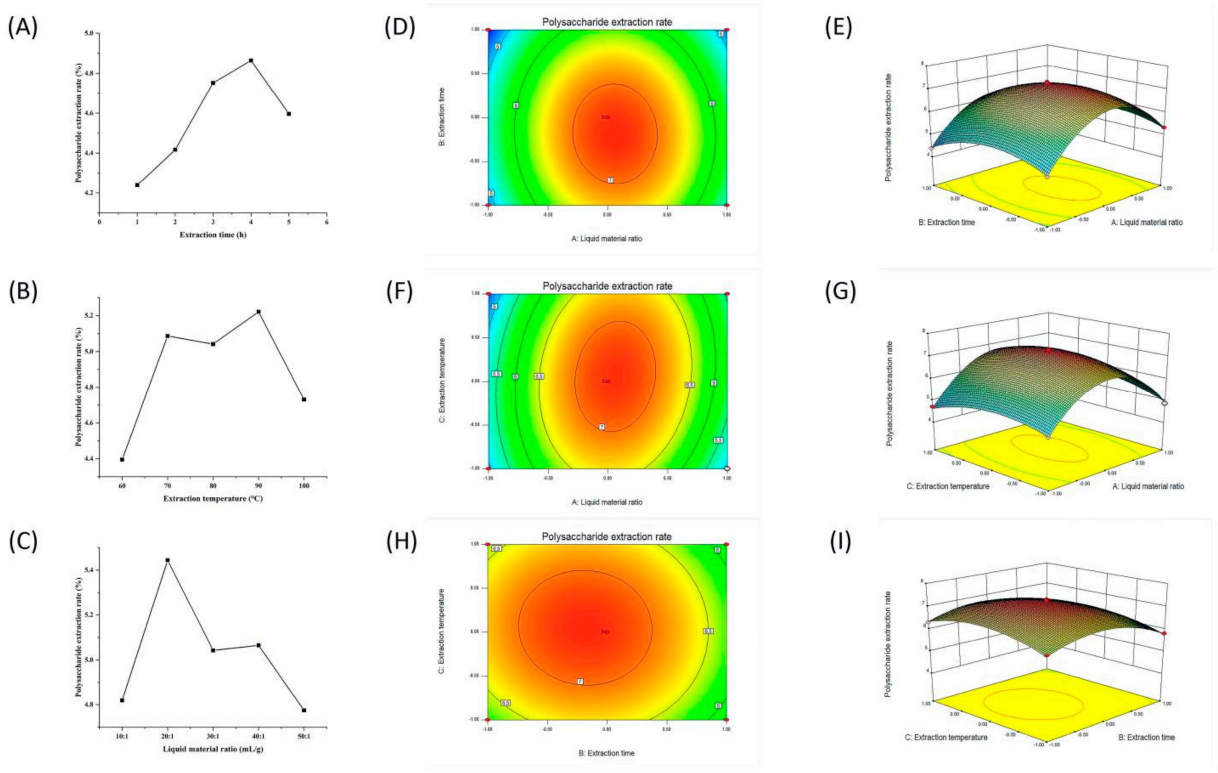

Fig. S1. The results of single factor test were about the effect of extraction time(A), extraction temperature(B), and liquid material ratio(C) on the polysaccharide from *Sinopodophyllum hexandrum* fruit extraction rate. Response surface diagram of the interaction between extraction time and liquid material ratio on the polysaccharide extraction rate(D-E). Response surface diagram of the interaction between extraction temperature and liquid material ratio on the polysaccharide extraction rate(F-G). Response surface diagram of the interaction between extraction time and extraction temperature on the polysaccharide extraction rate(H-I).

As shown in Fig. S1(A), we fixed the extraction temperature and liquid material ratio. The extraction time remained unchanged. When the extraction time reached 4 h, the polysaccharide extraction rate reached the highest value of 4.864%, and with an increase in time, the polysaccharide extraction rate began to decline. Fig. S1(B) shows the influence of the extraction temperature on the extraction rate of polysaccharides. When the extraction time and liquid material ratio were fixed, the highest extraction rate of 5.221% was obtained at 90°C. According to Fig. S1(C), the extraction time and temperature were kept

unchanged, and only the liquid material ratio was changed. When the liquid material ratio was 20:1, the extraction rate of the polysaccharide reached a maximum of 5.444%.The response surface software Design ExpertV8.0.6.1 was used to design the three-factor and three-level tests. The design scheme and results are shown in Table S3, and the regression model variance analysis is shown in Table S4. The three factors were regressed and fitted with the extraction rate of the polysaccharide, and the regression equation is as follows[3]:

$Y=7.19+0.20A–0.24B+0.042C–0.056AB+0.2AB–0.039BC–1.71A^2–0.67B^2–0.49C^2$

Correction coefficient R^2Adj = 0.9874, predicted complex correlation coefficient R^2=0.9945, and predictive correlation coefficient R^2(Pred) = 0.9685 are close to the predicted complex correlation coefficient [4], indicating that the fitting degree was good. Lack of fit P = 0.6827 > 0.05 shows no significant difference. The coefficient of variation (CV%) is 2.04%, indicating that the model selected fitted the test well[5]. The order of the influence degree of the three factors was extraction time > liquid material ratio > extraction temperature. The degree and significance of the interaction between several factors can be reflected by the three-dimensional image and contour line of the response surface. The steeper the slope of the three-dimensional image and the closer the contour map was to the ellipse, the difference was significant. As shown in Fig. S1(D-I), the three-dimensional curve slope of liquid material ratio (a) and extraction temperature (c) is the steepest, and the ovality of the high line is the largest, followed by the liquid material ratio (a) and extraction time (b), and finally the extraction temperature (c) and extraction time (b). It can be concluded that the degree of influence on the interaction of two factors on the extraction rate of the polysaccharide was ac > ab > bc. The optimal scheme obtained by the software is as follows: a is 20.66:1 (mL/g), b is 3.81 h, c is 90.65°C, and the predicted value of extraction rate was 7.216%. To improve the operability of the test, a was set to 20.7 (mL/g), b to 3.8 h, and c to 90.7°C, and

three parallel tests were conducted. The result was 7.066%, which was close to the predicted value, which proved the effectiveness of this model [6].

Table S3 Results of polysaccharide response surface test

Serial number	Liquid material	Extraction	Extraction	Extraction rate of
1	0	0	0	7.319
2	0	1	-1	5.824
3	0	-1	-1	6.248
4	1	-1	0	5.333
5	-1	0	1	4.708
6	0	0	0	7.208
7	0	1	1	5.734
8	1	1	0	4.752
9	0	0	0	7.275
10	0	0	0	7.141
11	-1	1	0	4.395
12	1	0	1	5.444
13	1	0	-1	4.864
14	0	-1	1	6.315
15	-1	0	-1	4.931
16	-1	-1	0	4.752
17	0	0	0	6.984

Table S4 Analysis of variance of response surface regression model

Sources of	Sum of	Degrees of	The mean	F	P	significant
Model	17.36	9	1.93	139.77	< 0.0001	***
A	0.32	1	0.32	23.40	0.0019	**
B	0.47	1	0.47	34.20	0.0006	**
C	0.014	1	0.014	1.01	0.3482	
AB	0.013	1	0.013	0.91	0.3721	
AC	0.16	1	0.16	11.68	0.0112	**
BC	0.006162	1	0.006162	0.45	0.5253	
A^2	12.32	1	12.32	892.85	< 0.0001	***
B^2	1.87	1	1.87	135.75	< 0.0001	***
C^2	1.00	1	1.00	72.74	< 0.0001	***
Residual	0.097	7	0.014			
Lack of Fit	0.028	3	0.009219	0.54	0.6827	
Pure Error	0.069	4	0.017			
Cor Total	17.45	16				

A is the liquid material ratio, B is the extraction time, and C is the extraction temperature. Significance analysis of differences $^{**}p \leq 0.05$, $^{***}p \leq 0.0001$.

References

[1] Dong, M., Jiang, Y., Wang, C., Yang, Q., Jiang, X., & Zhu, C. (2020). Determination of the Extraction, Physicochemical Characterization, and Digestibility of Sulfated Polysaccharides in Seaweed-Porphyra haitanensis. Marine drugs, 18(11), 539. https://doi.org/10.3390/md18110539

[2] Wang, J., Chen, X., Gao, W., Zhang, Z., Zhang, Y ., & Liu, T. (2022).Optimization of ultrasonic-assisted extraction process and physiological activity of polysaccharide from Dendranthema morifolium by response surface methodology. China Food Additives(02),100-109. doi:10.19804/j.issn1006-2513.2022.02.014.

[3] Bojanić, N., Fišteš, A., Rakić, D., Kolar, S., Ćurić, B., & Petrović, J. (2021). Study on the effects of smooth roll grinding conditions on reduction of wheat middlings using response surface methodology. Journal of food science and technology, 58(4), 1430–1440. https://doi.org/10.1007/s13197-020-04654-5

[4] Es-Haghi, A., Taghavizadeh Yazdi, M. E., Sharifalhoseini, M., Baghani, M., Yousefi, E., Rahdar, A., & Baino, F. (2021). Application of Response Surface Methodology for Optimizing the Therapeutic Activity of ZnO Nanoparticles Biosynthesized from Aspergillus niger. Biomimetics (Basel, Switzerland), 6(2), 34. https://doi.org/10.3390/biomimetics6020034

[5] Ji, Y. B., & Wang, F. L. (2021). Optimization of trypsin extraction technology of Allium cepa L. polysaccharide by response surface methodology and the antitumor effects through immunomodulation. Bioengineered, 12(1), 382–391.

[6] Li, F., Gao, J., Xue, F., Yu, X., & Shao, T. (2016). Extraction Optimization, Purification and Physicochemical Properties of Polysaccharides from Gynura medica. Molecules (Basel, Switzerland), 21(4), 397. https://doi.org/10.3390/molecules21040397
